# Impact of the physical therapy–managed spinal orthoses program on cost of care in the hospital setting: a retrospective interrupted time-series study

**DOI:** 10.1093/intqhc/mzac094

**Published:** 2022-11-23

**Authors:** Sue Willey, James Lenk, Linda Waters, Charles Joseph French, Jonathan Mathew Cayce

**Affiliations:** Physical Therapy, Ascension Via Christi, 929 N. St Francis St, Wichita, KS 67214, USA; Physical Therapy, Ascension Via Christi, 929 N. St Francis St, Wichita, KS 67214, USA; Physical Therapy, Ascension Via Christi, 929 N. St Francis St, Wichita, KS 67214, USA; DeRoyal Industries, 200 DeBusk Lane, Knoxville, TN 37849, USA; DeRoyal Industries, 200 DeBusk Lane, Knoxville, TN 37849, USA

**Keywords:** quality improvement, off-the-shelf spinal orthoses, economic evaluation, patient outcomes, centralized process, physical therapy

## Abstract

**Background:**

The physical therapy (PT) department at a level 1 trauma center identified vendor delivery delays of off-the-shelf (OTS) spinal orthoses that delayed patient mobilization.

**Objective:**

This study aimed to identify improvements in mobilization times, discharge times and reduction in the cost of care after centralizing the management of orthoses within the therapy department.

**Method:**

The centralized management of OTS spinal orthoses included stocking three adjustable lumbosacral and thoraco-lumbosacral orthosis sizes and ensuring that all personnel received training to appropriately fit the orthoses to patients. This study evaluates the impact of the centralized program by using a retrospective interrupted time-series design to compare outcomes before and after program implementation. Outcome measurements included orthosis delivery delay, time to orthosis delivery, time to mobilization by physical therapist, length of stay (LOS) and cost of care. Segmented linear regression, Wilcoxon rank-sum test and Fisher's exact tests compared outcome measures before and after implementing the centralized program.

**Results:**

The PT-managed program eliminated orthosis delivery delays noted during the vendor program (42 vs. 0; *P* < 0.001), resulting in an overall 13.97-h reduction in time to mobilization (*P* < 0.001). Program cost savings equated to $2,023.40 per patient (*P* < 0.001). Sub-group analysis of patients without complications and treated conservatively showed a significant reduction in LOS (15.36 h; *P* = 0.009) in addition to time to mobilization reductions.

**Conclusion:**

The PT-managed program significantly improved the quality of care for patients who required a spinal orthosis by mobilizing patients as soon as possible, allowing timely discharge. The program also resulted in overall patient and hospital cost savings.

## Background

Surgical care and non-surgical care of hospitalized patients with spinal fractures, traumatic spinal injuries and spine disorders have progressed. Standard practice once confined patients to extensive bed rest, but research shows improved outcomes with earlier mobilization with and without spinal orthoses [1–7]. Initial spine bracing utilized body casting and custom orthoses; however, recent research has shown that ‘Off-the-Shelf’ (OTS) adjustable orthoses benefit patients in their recovery [3–5, 8–11]. One study indicates that OTS orthosis use for Medicare patients can save up to $2000 in healthcare costs over the 18 months following spinal injury. Furthermore, the research credits part of the savings to initial physical therapy (PT) interventions, including teaching patients the correct use of their orthoses [3].

Hospitals are increasingly under economic pressure to provide excellent patient care with less financial resources. Many factors influence the cost of hospitalization for patients with spinal fractures. Length of stay (LOS) contributes significantly to hospital costs. Hospital days account for up to 86% of the total cost for non-operative spinal injuries and 48% for surgical patients [12]. Delays in care, such as orthosis delivery delays, negatively impact the LOS. Patients must frequently remain immobile until a clinician fits them with a spinal orthosis. A delay in orthotic fitting prolongs immobility, postpones initial mobilization with PT and increases the LOS [6].

Many facilities outsource spinal orthosis fitting and delivery to external vendors. At the study facility, ordering orthoses from a vendor involved several steps. First, the physician enters orders for a thoracolumbar sacral orthosis (TLSO) or a lumbar sacral orthosis (LSO) in the electronic medical record. The nurse or healthcare unit clerk then completes a paper order and faxes it to the vendor with all the supporting documents, such as patient demographics, insurance information, patient’s height and weight and a copy of the order. The study facility frequently lacked communication between the facility and the vendor, leading to notable service delays. The vendor did not consistently communicate order receipt, expected orthosis delivery time or needed additional information.

In response to vendor delivery issues, an interdisciplinary team involving PT, spine and trauma physicians, nursing and the supply chain began a quality improvement project to address the identified problems. The project instituted our PT-managed spinal orthoses program that allows immediate fitting and mobilization of patients upon physician order. This study evaluates how the quality improvement project impacted patient care. The primary objective aimed to characterize changes in the LOS pre- and post-implementation of the PT-managed spine orthosis program. Secondary objectives included evaluation of time to mobilization, time to spinal orthosis delivery and process-related care costs.

## Methods

### Study facility

A level I trauma center in Wichita, Kansas, USA, serves over 19 000 patients annually. Patients receive spinal orthoses as inpatients following traumatic injury or spinal surgery. The hospital institutional review board approved the study protocol and waived the informed consent requirement.

### Study design

We evaluated the impact of the PT-managed spinal orthoses program using a retrospective interrupted time-series design [13–15]. The study population consisted of patients who received an LSO or TLSO during their inpatient care as defined by the intervention period. The pre-intervention period occurred between January 2018 and February 2019. A record review identified 100 patients who received a spinal orthosis in this period to comprise the vendor group. The post-intervention period occurred between February and August 2019, and the record review identified 120 patients forming the PT group. Table 1 summarizes the demographics, and Table 2 summarizes the patients’ diagnoses for both groups.

**Table 1:** Demographics of patients who received an LSO or TLSO during the study period

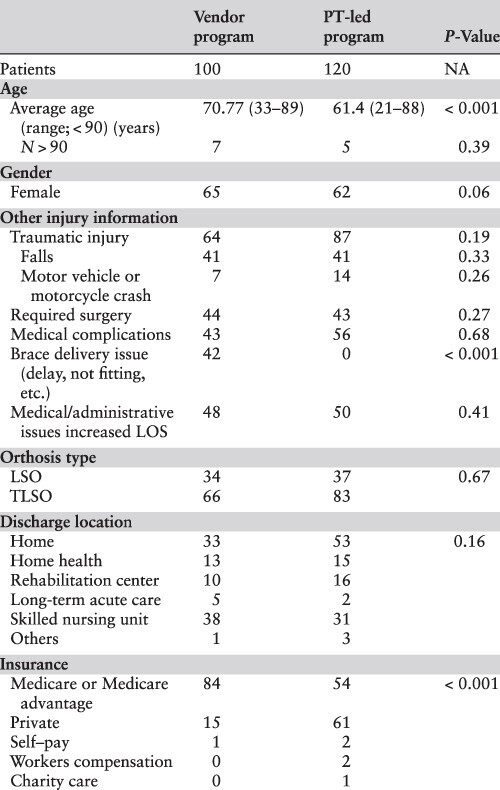

**Table 2:** Primary, secondary and tertiary diagnoses information of patients included in the study

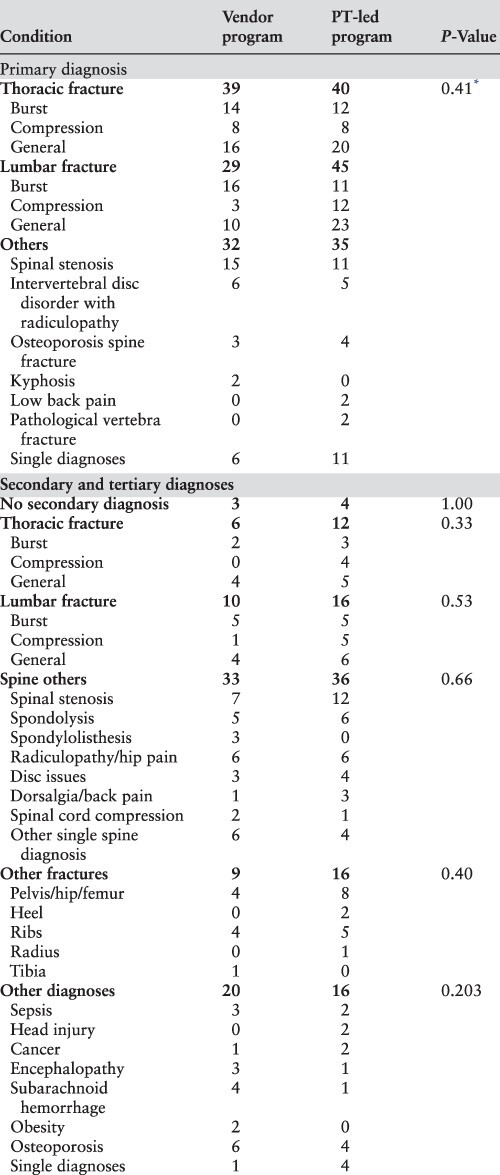

* P-value results from comparing thoracic, lumbar and other categories.

The bold represents the primary injury type. Each subentry below represents sub type of injury.

### Development of the PT-managed spinal orthoses program

An interdisciplinary task force led by physical therapists involving physicians nursing, and supply chain was formed to address the identified challenges. Supply chain sourced OTS spinal orthoses from contracted medical supply vendors. The team assessed sourced OTS orthoses to determine the orthosis that best met the patients’ needs, leading to selecting an orthosis that allowed customization for LSO and TLSO. This selection allowed the hospital to stock one device in three sizes with accessories to address all relevant thoracic and lumbar spinal conditions.

Representatives from the OTS orthosis manufacturer met with the PT leadership to discuss implementing a centralized orthoses program where physical therapists provide and fit patients with LSO and TLSO orthoses. Physical therapists proposed this program to the task force and received approval. The PT department received in-servicing for fitting, donning and doffing, use and care of the orthoses. The PT department disseminated the training to nursing staff to help facilitate patient education and ensure that nursing could aid in patient mobilization. A physical therapist preceptor checked off staff members for competencies. Supply chain, information technology and the OTS manufacturer worked with physical therapists to implement a process to optimize inventory levels, streamline the reordering process and digitize the ordering and billing process. Physical therapists educated the physicians and nurses on the new process for ordering spinal orthoses from the PT department during the first week of February 2019.

### Data analysis

Researchers extracted basic demographic information and study-related variables from the hospital’s electronic health records. Study variables included diagnosis including traumatic injury or need for surgery, brace type, brace cost, PT service cost, total case cost, insurance/payer type, discharge location, complications experienced, delays with a cause in orthosis delivery or mobilization and the following dates and times: admission, orthosis order, mobilization and discharge. The research team utilized the dates and times to investigate the time from admission to orthosis delivery, mobilization and LOS. In the absence of vendor delivery times, we conservatively estimated vendor orthosis delivery based on the time of order, assuming that the vendor delivered at 12:00 or 17:00. Any bias introduced by this estimate benefitted the vendor program over the centralized spinal program. Researchers performed overall and subgroup analyses (e.g. surgery vs. trauma) to investigate how the program impacted the care of specific groups.

All data analysis was performed in the R statistical software using the RStudio user environment [16, 17]. Continuous metrics were analyzed utilizing segmented linear regression to model differences between the two programs and visualize how the variable changed during each intervention period [13–15]. The procedure used for the segmented linear regression utilized the ITS analysis R package that uses Type II sum squares analysis of covariance-lagged dependent variable to determine significant differences between the two programs [18] Where appropriate, the modeled difference between programs is reported with a 95%CI. Demographic normal continuous data were analyzed with an independent *t*-test, non-normal data with the Wilcoxon rank-sum test, and Fisher’s exact test was used for categorical data. The analyst adjusted resultant *P*-values using the Holm–Bonferroni Method for each subgroup. An adjusted *P*-value < 0.05 was considered significant for this study.

## Results

The study identified 100 patients for inclusion in the pre-intervention period and 120 patients in the post-intervention period. Tables 1 and 2 show that all variables and diagnoses were similar between groups except for age, insurance type and incidence of orthosis delivery delay. Observed differences in age and insurance type resulted from documentation insufficiencies from the vendor for non-Medicare patients. We noted the program’s elimination of orthosis delivery delays (42 vs. 0; *P* < 0.001), justifying changing the orthosis fitting process. Similar diagnosis, fall and motor vehicle/ motorcycle crash rates between programs suggest comparable patient populations.

**Figure 1:**
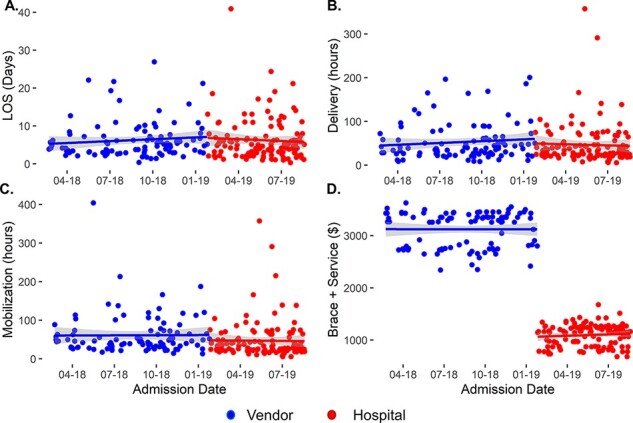
Segmented linear regression for the study population and key metrics: (A) LOS, (B) time to delivery of orthosis from admission, (C) time to mobilization from admission and (D) charges associated with providing orthosis and initial PT mobilization session. Changes in slope and *y*-intercept between intervention periods indicate differences between groups. The regression line indicated by the solid line, and shading indicates a 95%CI of the model.

Figure 1 summarizes the LOS, time to mobilization and delivery from admission, and the cost to provide the brace for all patients included in the study. Segmented regression shows that LOS, delivery of the orthosis and time to mobilization each decreased (Figure 1A-C), but only time to the mobilization of the patient indicated a significant difference (13.97 h; 95%CI: 0.17 to 27.77 h; *P* < 0.001). Patient charges for the orthosis, fitting and mobilization (Figure 1D) demonstrate a clear decrease under the PT-managed program ($2023.40; 95%CI: $1950.17 to $2096.64; *P* < 0.001).

**Figure 2:**
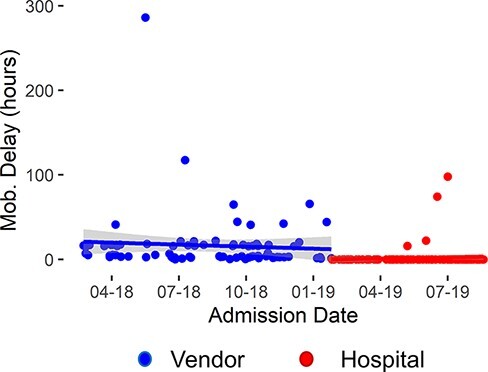
Mobilization delay after delivery of orthosis for all patients. Metric visualizes mobilization delays that negatively impacted patient care during the vendor-run program. The solid line indicates the regression line; shading indicates the model’s 95%CI.

The variability in the data visualized in Figure 1A-C reflects the study population differing acuity. Forty-five percent of patients experienced a discharge delay due to medical or administrative issues. Outliers in the pre- and post-intervention periods skewed modeled results. We normalized the data by calculating the time difference between orthosis delivery and patient mobilization, resulting in mobilization delay (Figure 2). Segmented regression of mobilization delays indicates that the centralized program reduced mobilization time by 14.75 h (95%CI: 7.8 to 21.6 h; *P* < 0.001). This finding supports the conclusion that the PT program mobilized patients sooner by providing and fitting spinal orthoses to our patients instead of waiting on a vendor.

**Figure 3:**
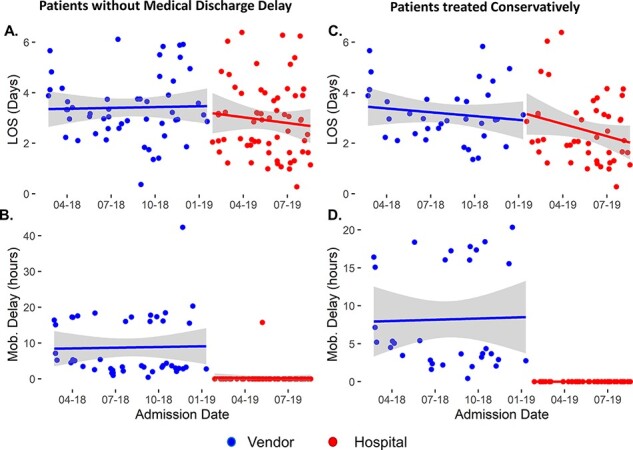
Program impact on the LOS and mobilization delays for patients that did not experience medically necessitated discharge delays (A and B) and patients who received only conservative treatment (C and D). In both subpopulations, segmented linear regression visualizes a slope change for both groups demonstrating the decreased LOS (A and C) and near elimination of mobilization delays with the Centralized Hospital Program. The solid line indicates the regression line, and shading indicates the 95%CI of the model.

Variable patient acuity prompted subpopulation analysis for patients hypothesized to experience the greatest benefit from the PT-managed program. These populations include those that did not experience medical discharge delays (Vendor = 52; PT = 63) and those that only required conservative treatment (vendor = 35; PT = 46). Figure 3A and B summarizes the segmented regression findings from the subpopulation that excluded patients with medical discharge delays. This subpopulation experienced a near significant 11.90 h (95%CI: −0.62 to 24.48 h; *P* = 0.070) reduction in LOS and an 8.32-h (6.06 to 10.59 h; *P* < 0.001) significant decrease in mobilization delay. Figure 3C and D displays outcomes for patients treated conservatively without surgery, demonstrating a significant reduction in LOS of 15.40 h (95%CI: 1.8 to 29.04 h; *P* = 0.03) and elimination of mobilization delays 7.95 h (95%CI: 5.97 to 9.93 h; *P* < 0.001). These results highlight the benefits realized by patients treated without complications or requiring only conservative treatment.

**Figure 4:**
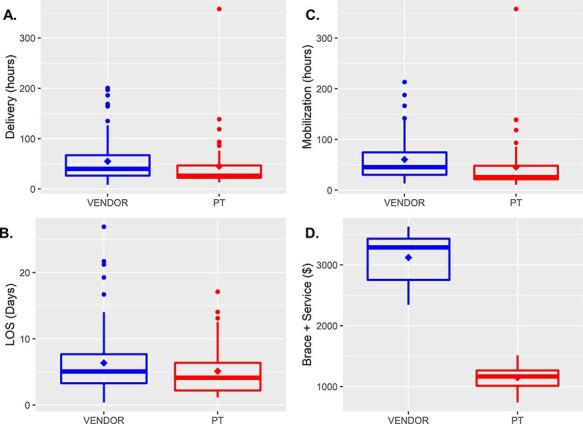
Centralized program positively impacts care for Medicare patients by ensuring timely orthosis delivery (A) and mobilization of patients (B). A noticeable decrease in LOS (C) occurred (30.47 h; *P* = 0.088). The centralized program decreased the cost per patient to provide the orthosis and mobilization (D) by $1972 (*P* < 0.001), resulting in significant Medicare savings. (A–D) The bold line represents the population median; the diamond represents the population mean; dots represent population outliers; the extent of the box indicates the 75th and 25th quartiles of the program population. The whisker extent represents the 1.5 × Inter Quartile Range (IQR) from the 25th and 75th percentiles.

Figure 4 summarizes outcomes for Medicare patients (vendor = 84; PT = 54). In the PT-managed program, the decrease in time to orthosis delivery approached significance (*P* = 0.054) with a modeled reduction of 9.67 h (95%CI: −6.72 to 26.06 h; Figure 4A). The PT-managed program significantly reduced the time to mobilization from admission (*P* < 0.001), equating to a modeled reduction of 14.72 h (95%CI: −1.53 to 30.95 h; Figure 4B). Medicare patients in the PT-managed program experienced a modeled LOS reduction of 30.47 h (95%CI: −5.57 to 66.51 h; *P* = 0.088; Figure 4C). Figure 4D shows a clear decrease in orthosis-related patient charges, equating to $1972.00 ($1870.97 to $2073.04; *P* < 0.001), demonstrating that the program collectively saved Medicare patients approximately $106 488 in orthosis-related charges.

**Figure 5:**
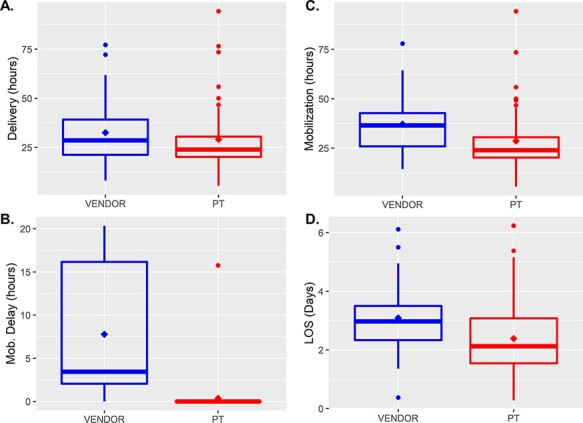
The Centralized Program facilitates the discharge of non-delayed patients home. Time to deliver an orthosis (A) became more consistent with centralization. Consistent delivery of the orthosis translated mobilization times (B) to decrease by 14.44 h (*P* = 0.004). Panel C demonstrates the variability in mobilization delays from inconsistent vendor delivery. Overall, patients discharged home experienced a 15.67-h reduction in LOS in the centralized program (*P* = 0.007). (A–D) The bold line represents the population median; the diamond represents the population mean; dots represent population outliers; the extent of the box indicates the 75th and 25th quartiles of the program population. The whisker extent represents the 1.5 × IQR from the 25th and 75th percentiles.

The final subpopulation investigated patients discharged home without a discharge delay (vendor = 35; PT = 44). This population represents patients who benefit from quick treatment and discharge. No difference existed between the two groups concerning orthosis delivery to the patient; however, Figure 5A shows less variability of orthosis delivery time from admission to the patient compared to the vendor. Figure 5B demonstrates a 14.44-h decrease in mobilization time in the PT program (95%CI: 7.89 to 20.98 h; *P* = 0.004). The mobilization delay (Figure 5C) visualizes how delay varied greatly during the vendor program compared to the near elimination of delays in the PT-managed program. Patients discharged home without delay in the PT-managed program experienced a reduction in LOS by 15.67 h (95%CI: 2.45 to 28.90 h; *P* = 0.007; Figure 5D).

## Discussion

### Statement of principal findings

Timely brace delivery and mobilization by PT correlate with decreased LOS, directly addressing a high cost to spinal injury patients [6, 12]. The results of this study show that the PT-managed program reduced mobilization delays (Figure 2) realized by timely brace delivery and mobilization of patients. This process improvement resulted in a trend toward shorter LOSs (Figure 1A) and near elimination of mobilization delays. The benefits of the PT-managed program became more evident in the analysis of the subpopulations that benefited most from the process improvement initiative, patients with no medically related discharge delays and those treated conservatively. Medicare patients benefited from a reduced LOS and a reduction in charges to Medicare. The program allowed the hospital to discharge patients without complications home 15.65 h sooner than the vendor program, allowing the PT department to manage orthoses inventory, fit the orthoses and proceed with mobility activities, often in the same visit, to improve the quality of care delivered to spinal injury patients. The facility, specifically the PT department’s ownership of the process, represented the key to reducing orthosis delivery and patient mobilization time. Maintaining orthoses onsite and training hospital clinicians to fit them intrinsically increased process convenience, naturally leading to more timely fitting, delivery and mobilization. Outcomes continued to improve as therapists became more experienced with the products, and communication improved within the hospital team and overall system.

### Strengths and limitations

This study followed an interrupted time-series design that allowed for assessing the new program as a function of time. Segmented linear regression accounts for seasonality, helping minimize the risk of erroneous conclusions. Furthermore, the study design and analysis techniques allow for identifying immediate and lagged changes. We observed immediate changes (step change) in cost of service and time to mobilization (Figure 1D and C); however, changes in the LOS lagged (slope change) program implementation (Figures 1A and 3A and C).

The interdisciplinary team approach allowed us to quickly implement the change and receive complete buy-in from our staff. The new program enabled the hospital to improve the quality of care to our patients while reducing care costs. Our program has extended to the emergency department (ED) allowing patients requiring only conservative treatment to avoid an overnight admission by receiving a spinal orthotic in the ED.

A limiting factor was the retrospective design of the study. Once data collection started, the research team realized that the vendor did not document orthosis delivery in the medical record, preventing an accurate comparison of the delivery time between the two groups. The analysis strategy changed to look at the date/time of mobilization from the admission date/time, knowing that this strategy would capture any delivery delays. We estimated that delivery delays by assuming a best-case delivery time for the vendor to bias result in favor of the vendor program. The inability to precisely determine the vendor’s delivery time minimizes the reliability of brace delivery time results. The results for time to mobilization from admission provide a more reliable measure.

### Interpretation with the context of the wider literature

Patients benefited most by earlier mobilization under the PT program. The literature demonstrates that early mobilization decreases the risks of complications associated with prolonged bed rest such as pneumonia, deep vein thrombosis and deconditioning [6]. Early mobilization allowed patients to begin rehabilitation as soon as possible, facilitating timely discharge from the hospital.

Patients discharged to home represent patients who do not need intensive rehabilitation, and these patients experienced a 15-h LOS reduction in the PT program. Decreasing the LOS for patients requiring minimal intervention helped reduce the risk of hospital-acquired conditions [18, 19].

Literature shows that orthoses help reduce the cost of medical care for Medicare patients after the hospital, possibly by keeping them more active [3]. Medicare requires specific documentation from durable medical equipment vendors to provide orthoses, and these patients can experience discharge delays due to requiring approvals, a common issue faced by Medicare patients [20–22]. The study results show that Medicare patients who benefited from the new orthosis delivery model experienced a 14.72-h reduction in time to mobilization (Figure 4A and B), leading to these patients becoming active sooner during their hospital stay. Increased activity in the hospital likely contributed to the 30-h average reduction in LOS. The hospital system reduced average orthosis-related costs by $1972 per patient. These benefits show how the program aided the hospital in achieving reduced cost and improved quality as mandated by the Centers for Medicare and Medicaid Services. Government-funded healthcare systems, common outside the USA, must still control costs and provide quality care to patients [23–27]. Translation of the program described in this paper may vary from country to country based on how the individual healthcare system or hospital utilizes spinal orthotics [27, 28]. For example, external vendors do provide spinal orthotic services to the government-funded healthcare system in the UK and Saudi Arabia. As such, hospitals in these countries could achieve similar benefits (earlier mobilization and reduced LOS) as described in this study.

Other countries, such as Germany and the Netherlands, do not commonly use OTS orthotics to conservatively manage spinal injuries or immobilize patients after spine surgery [26, 27]. However, these healthcare systems could still benefit from a similar program. Government-funded hospitals could evaluate impediments to early patient mobilization and hospital discharge following spinal injury. Such an evaluation may identify similar impediments, e.g. inability to safely immobilize the spine to protect the patient, or identify new impediments to quality patient care [29]. Either way, eliminating the identified impediments to early immobilization will improve the quality of care provided to the patients as demonstrated in this study.

### Implications for policy, practice and research

Prioritization by the vendor on when to deliver and fit orthoses limited the physical therapists’ ability to mobilize the patient and led to the creation of the PT-led program. The new program eliminated mobilization delays related to orthosis delivery because we maintained orthoses on-site. With the PT program, mobilization of patients occurred in conjunction with orthosis delivery and fitting, eliminating mobilization delays. Physical therapists could respond to orders quickly with on-site availability, resulting in quicker mobilization of patients, shorter LOSs and decreased costs to the patient.

Using an outside vendor for OTS spinal orthoses led to unnecessary costs to the hospital and patients. The vendor charge matched the maximum Medicare allowable, a charge the hospital had to absorb. The new program realized immediate cost savings. Reductions in LOS contribute to program-related cost savings. Delays in orthosis delivery, mobilization and insurance approvals unnecessarily prolonged patient hospitalization causing increased costs to the patient [30–33].

Hospitals constantly try to improve efficiencies in patient flow by implementing programs to reduce ED wait times, facilitate rapid admission to patient care units and improve the timeliness of discharges to maximize utilization of hospital beds and minimize patient wait time for a bed [34, 35]. This study shows how a relatively small change, transitioning the process to provide patient spinal orthoses from an external vendor to an internal hospital department, can substantially impact key hospital efficiencies. Continuous improvement efforts led to the PT department providing the orthoses to other areas of the hospital system. Most notably, the program has prevented admissions by providing orthoses in the ED. The centralized service now represents the standard of care, and the PT-led program has expanded to three additional acute care hospitals and the system’s rehabilitation hospital.

Qualitative improvements included improved timeliness of spinal orthoses fitting, increased ease of obtaining service and improved communication and satisfaction among hospital personnel. The PT department fitted and mobilized patients with spinal orthoses as soon as providers placed orders. The new process allowed care teams to address any fit issues immediately. Process improvements improved direct communication between nurses, physicians and physical therapists, resulting in improved satisfaction with providing spinal orthoses to patients.

In this study, we demonstrate how internalizing a service previously provided by an outside vendor improved care quality and reduced costs. At the onset of this project, the team knew that the program would realize per-patient cost savings and enhance the quality of care by ensuring orthosis availability. Other care processes outsourced to a vendor or associated with unnecessary care could experience benefits in care quality and cost savings by implementing similar quality improvement initiatives [33, 36, 37].

## Conclusions

This quality improvement initiative allowed the PT department to improve quality and reduce the cost of care. The new process allowed the care team to mobilize patients more quickly and facilitated more timely discharge. Centralizing the spinal orthosis process resulted in significant cost savings for the hospital and the patient. Ultimately, the spinal orthoses program became the standard of care in multiple hospital locations in the study hospital’s system.

## Data sharing statement

The data underlying this article will be shared upon reasonable request to the corresponding author.
